# Ubenimex combined with Albendazole for the treatment of *Echinococcus multilocularis*-induced alveolar echinococcosis in mice

**DOI:** 10.3389/fvets.2024.1320308

**Published:** 2024-03-15

**Authors:** Zhen Zhou, Meiduo Huayu, Yalin Mu, Feng Tang, Ri-Li Ge

**Affiliations:** ^1^Research Center for High Altitude Medicine of Qinghai University, Xining, Qinghai, China; ^2^Key Laboratory of High Altitude Medicine in Qinghai Provincial, Qinghai University, Xining, Qinghai, China; ^3^Department of Medical Imaging Center, Qinghai University Affiliated Hospital, Xining, Qinghai, China

**Keywords:** LAP, Ubenimex, Albendazole, *E. multilocularis*, liver, mice

## Abstract

**Introduction:**

Alveolar echinococcosis (AE) is a parasitic disease caused by *E. multilocularis metacestodes* and it is highly prevalent in the northern hemisphere. We have previously found that vaccination with *E. multilocularis*-Leucine aminopeptidase (EM-LAP) could inhibit the growth and invasion of *E. multilocularis* in host liver, and Ubenimex, a broad-spectrum inhibitor of LAP, could also inhibit *E. multilocularis* invasion but had a limited effect on the growth and development of *E. multilocularis*.

**Methods:**

In this study, the therapeutic effect of Ubenimex combined with Albendazole on AE was evaluated. Mice were intraperitoneally injected with *protoscoleces* and imaging examination was performed at week 8 and week 16 to detect cyst change. During this period, mice were intraperitoneally injected with Ubenimex and intragastrically administered with Albendazole suspension. At last, the therapeutic effect was evaluated by morphological and pathological examination and liver function.

**Results:**

The results revealed that the combined treatment could inhibit the growth and infiltration of cysts in BALB/c mice infected with *E. multilocularis protoscoleces*. The weight, number, invasion and fibrosis of cysts were reduced in mice treated with Ubenimex in combination with Albendazole. The same effect was achieved by the single Ubenimex treatment because of its inhibitory effect on LAP activity, but it was less effective in inhibiting the growth of cysts. The levels of ALT, AST, TBIL, DBIL, ALP, and γ-GT were reduced after the combined treatment, indicating that treatment with both Ubenimex and Albendazole could alleviate liver damage.

**Discussion:**

This study suggests that the combined treatment with Ubenimex and Albendazole could be a potential therapeutic strategy for *E. multilocularis* infections.

## Introduction

Alveolar echinococcosis (AE) is a zoonotic parasitic disease caused by *Echinococcus multilocularis metacestodes* and it is highly prevalent in the northern hemisphere, particularly in developing countries (1). AE is difficult to treat and can only be removed surgically in clinic. However, not all infection foci could be removed surgically in end-stage AE patients with multiple organ metastasis, and in this case liver transplantation becomes the only therapeutic strategy (2). Drug therapy still plays a major role in the treatment of AE (3). Imidazole derivatives, such as Albendazole and Phenimidazole, are included in the World Health Organization's (WHO) Standard List of Essential medicines, but they have a limited effect on AE as they could only inhibit the uptake of glucose and deplete the glycogen of the germinal layer cells but could not restrain the growth and invasion of parasites (4, 5). *E. multilocularis protoscoleces* grow like a malignant tumor in the host and will infect the whole liver within only 5–10 years. AE may develop into a “parasitic cancer” and metastasize to other organs, causing portal hypertension, cerebral echinococcosis, pulmonary echinococcosis and even death (4, 6). Therefore, AE has become a public health problem of worldwide concern, especially in some developing countries such as China, Algeria, Kazakhstan and Mongolia. This highlights the need to find effective ways to control the growth and invasion of parasites (4). The difficulty in treating AE arises from the insidious onset and infiltrative growth and the tiny lesions are likely to relapse. *E. multilocularis metacestodes* and cysts can invade the vasculature, resulting in metastases to other liver lobes and even other tissues and organs. *E. multilocularis* can also lead to liver fibrosis through *E. multilocularis* Leucine aminopeptidase (EM-LAP) (7, 8). Existing drugs like Albendazole have lower water solubility and drug concentration in hydatid lesions, making it difficult to completely clear all parasites and restrain infiltrative growth (9). Even if increase the concentration or prolonged the treatment periods of Albendazole, may not improve treatment effect, but some toxic effect such as liver toxicity, allergic reactions, and rarely severe myelosuppression may occur (10). Up to now, there still has not ideal therapeutic methods and medicines to heal it, surgical operation and organ transplantation was only option in end-stage of AE.

Some other research has reported that drug combination was an effective way to treat AE, the combination of Carvacrol and Albendazole can enhanced the efficacy of monotherapy in AE, this therapeutic method may allow to improve the efficacy of the treatment without increasing the doses of Albendazole or prolong the therapy period, reducing the occurrence of toxic effects and adverse effects (11). Metformin can improve the therapeutic effect of low-dose Albendazole against AE, combination therapy with Metformin and Albendazole can reduce the toxicity of the high-dose Albendazole monotherapy currently employed, and increase the efficacy of the treatment (12). Albendazole combined the Isethionate/Hypromellose Acetate Succinate can improvement of the bioavailability and anti-hepatic AE effect (13). Therefore, a potential therapeutic strategy is to combine different drugs to reduce or inhibit the infiltrative and invasive growth of *E. multilocularis*.

We have previously found that vaccination with EM-LAP induced specific immune response and inhibited the invasion of *E. multilocularis* (7). We have also evaluated the therapeutic effect of recombinant EM-LAP (rEM-LAP) on AE and found that Ubenimex, a broad-spectrum inhibitor of LAP, also showed similar inhibitory effect on the invasion of *E. multilocularis* (8). These studies suggest that EM-LAP could be a potential therapeutic target of *E. multilocularis* infection. LAP is a metalloprotease of the M17 family (14) and it plays a crucial role in many physiological processes including growth, nutrition and metabolism. It is especially important for the invasion of pathogenic parasites in the host (15). There is also evidence that vaccination with Fasciola hepatica leucine aminopeptidase induced specific IgG antibody responses against infection, invasion and migration of Fasciola hepatica in the host (16). LAP also acts as a therapeutic target in Malaria infection (17). A number of studies have reported that LAP inhibitors such as Ubenimex (18) and CHR-2863 (19) could inhibit the enzymatic activity of Malaria M17 leucine aminopeptidase (PfM17LAP) and thus effectively decreased the digests host cell hemoglobin and invasion growth (19–21). Our previous studies have also found that Ubenimex had inhibitory effect on the invision of *E. multilocularis* in host liver but little effect on the nutrition, metabolism and growth of the parasites. Thus, the purpose of this study is to find an effective treatment for *E. multilocularis* infection.

Ubenimex is a broad-spectrum inhibitor of LAP and it is often used as adjuvant therapy for malignant tumors (22, 23). Ubenimex can inhibit tumor aggressiveness and development through recede the aminopeptidase CD13 (24, 25), and it also plays an important role in the treatment of many parasitic diseases such as Malaria (26) and *E. multilocularis* infection (8, 27) because of its ability to reduce the invasiveness and digestion of LAP in the host. Albendazole is a well known anthelmintic that can inhibit many parasitic infections (28), and it also acts as a suppressor of glycogen metabolism in parasites that leads to nutritional failure and death, but it is less effective for *E. multilocularis* infection (29). However, there is evidence that Albendazole in combination with other drugs may be more effective in inhibiting parasite invasion and growth (30). Albendazole is effective in inhibiting the growth and development of *E. multilocularis* by inhibiting parasite's absorption of glucose that may lead to glycogen depletion or by inhibiting the fumarate reductase system that may lead to reduced production of ATP. However, it has a low drug concentration in hydatid lesions and therefore little inhibitory effect on the infiltrative growth of *E multilocularis*. Ubenimex can inhibit the invasion of *E. multilocularis metacestodes* in host liver by inhibiting LAP production and activity, but it has a limited effect on the growth and development of *E. multilocularis*. Thus, a better therapeutic outcome is expected by combining Ubenimex and Albendazole.

In this study, Ubenimex and Albendazole are used in combination in the treatment of the infection of *E. multilocularis protoscoleces*, which may provide new insights into the treatment of AE.

## Materials and methods

### Mouse model and treatment

All animal experiments were performed in compliance with the regulations of the Ministry of Science and Technology of China and approved by the Experimental Committee of Qinghai University (QHDX-2019-09). Male BALB/c specific pathogen free (SPF) mice of 6–8 weeks old were purchased from Beijing Spaefer Biotechnology Company (SCXK2019-0010) and fed with sterilized feed and water over a 24 h day/night cycle in an animal biosafety level II (ABSL-2) laboratory (7, 8, 31). All mice were intraperitoneally injected with 2,000 protoscoleces, which were isolated from Gerbillinae infected with *E. multilocularis* for over 20 weeks in the Research Center for High Altitude Medicine (7, 32). At week 8, mice were randomized into four groups of nine mice each, including Combination group (Ubenimex + Albendazole), Model group, Ubenimex group and Albendazole group (Table 1), and the first imaging examination (Ultrasonography and MRI) was performed. Mice received different treatments until 16 weeks and the second imaging examination was performed. After that, mice were sacrificed to evaluate the infection, growth and invasion of *E. multilocularis*.

**Table 1:** Animal groups and treatment.

**Group (*n* = 9)**	**Treatment**			
	**Durg**	**Method of administration**	**Dosage**	**Times**
Ubenimex group	Ubenimex with cosolvent	Intraperitoneally injected with Ubenimex	7.5 mg/kg/day	8–9 weeks
Albendazole group	Albendazole with 1% sodium carboxymethylcellulose	Intragastrically administered with Albendazole suspension	0.2 ml/20 g/day	8–9 weeks
Combination group	Both the Ubenimex and Albendazole	Intraperitoneally injected with Ubenimex and intragastrically administered with Albendazole suspension	7.5 mg/kg/day Ubenimex; 0.2 ml/20 g/day Albendazole	8–9 weeks
Model group	Only cosolvent	Intraperitoneally injected and intragastrically administered with cosolvent	Same dosages as treatment groups	8–9 weeks

A 9.13 mg of Ubenimex (Macklin Bio.; CAS: 58970-76-6) was dissolved in a solution consisting of 1,100 μl of DMSO, and then 4,400 μl of PEG300, 50 μl of Tween80, and 4,950 μl of normal saline were added (8, 33). Three gram of Albendazole (TCI Shanghai Chemical Industry Development Co. LTD.; CAS: 54965-21-8) was suspended in 1% sodium carboxymethylcellulose (11).

Treatment was initiated 4–6 weeks after protoscoleces inoculation. Mice in the Combination group were intraperitoneally injected with 7.5 mg/kg/day Ubenimex and intragastrically administered with 0.2 ml/20 g/day Albendazole suspension; mice in the Ubenimex group were intraperitoneally injected with only 7.5 mg/kg/day Ubenimex (8, 33, 34); mice in the Albendazole group were intragastrically administered with only 0.2 ml/20 g/day Albendazole suspension (11, 35, 36); while mice in the Model group only received pharmaceutical solvent (Table 1). All mice were treated for 8–9 consecutive weeks. At week 8 and week 16 after protoscoleces inoculation, imaging examination (Ultrasonography and MRI) was performed to observe the therapeutic efficiency and samples used for the comparison of therapeutic efficiency were taken from the same mice.

### Ultrasonography and MRI

Mice were anesthetized with 1.5% isoflurane in O_2_ on a specially designed heated bed for measurement of the cyst area in the epigastrium. Ultrasonography was performed using a FUJIFILM Vevo^®^ Ultrasonic imager for small animals; and MRI was performed using a BioSpec^®^ MRI scanner (Bruker PharmaScan 70/16 USR) and 7.0T MR Scanning system for small animals, which was equipped with a set of RF RES 300 1H 075/040 QSN TR application-specific radiofrequency mouse body coil for *in vivo* imaging. The body surface projection of the liver was positioned as close as possible to the center of the coil, and the height of the mouse in the coil was adjusted to ensure the liver at the center of the magnetic field for measurement of cysts in the liver and adjacent tissues. Physiological signals, such as breathing rate, were monitored throughout the experiment. The whole acquisition monitor process is triggered by breath gating. The scanning sequence was T1WI: TE 3.0 ms, TR 300.0 ms, turning Angle 90°, layer thickness 1.0 mm, FOV 35 mm × 35 mm, Averages = 6; T2WI: TE 25.0 ms, TR 2,000.0 ms, layer thickness 1.0 mm, FOV 35 mm × 35 mm, Averages = 16; DWI: TE 30.0 ms, TR 1,000.0 ms, b value 600 mm/s, layer thickness 1.0 mm, FOV 35 mm × 35 mm, Averages = 16.

### Morphological and pathological examination

At week 17 after the second imaging examination, all mice were euthanized and serum was separated from blood samples. The cysts of metacestodes in the liver and peritoneum were photographed to assess morphological alterations, and the weigh and number of cysts in the abdomen were also measured. Liver samples were optimal cutting temperature (OCT) embedded, sectioned into 4.5 μm-thick sections by a freezing microtome, and then stained with hematoxylin–eosin (Beijing Jiuzhoubailin Biotechnology Co. LTD.), Masson trichrome (Masson Trichrome Stain Kit Solarbio G1340) and Gomori-aldehyde-fuchsin (Modified Gomori-Aldehyde-Fuchsin Stain Kit Solarbio G1593) for histopathological examination of the invasion of metacestodes to adjacent tissues and the fibrosis level. Images were captured using a microscope (OLYMPUS MX63L) and the positive areas were identified using ImageJ software. The levels of alanine aminotransferase (ALT), aspartate amino transferase (AST), total bilirubin (TBIL), direct bilirubin (DBIL), albumin (ALB), alkaline phosphatase (ALP), and gamma-glutamyl transferase (γ-GT) in the serum were detected using a fully automatic biochemical analyzer (Chemray 800, Rayto Life and Analytical Sciences Co., Ltd.) to determine liver damages of mice in each group.

### Statistical analyses

All statistical analyses were performed using SPSS 22.0 and Microsoft Excel 2019. All measurement data were expressed as mean ± standard deviation ($\overset{\bar{\;}}{x}$ ± SD). Comparison between two groups was performed using the independent sample *T*-test; while that among multiple groups was performed using the one-way analysis of variance (ANOVA). *P* < 0.05 was considered statistically significant.

## Results

### Ultrasound and MRI results

**Figure 1:**
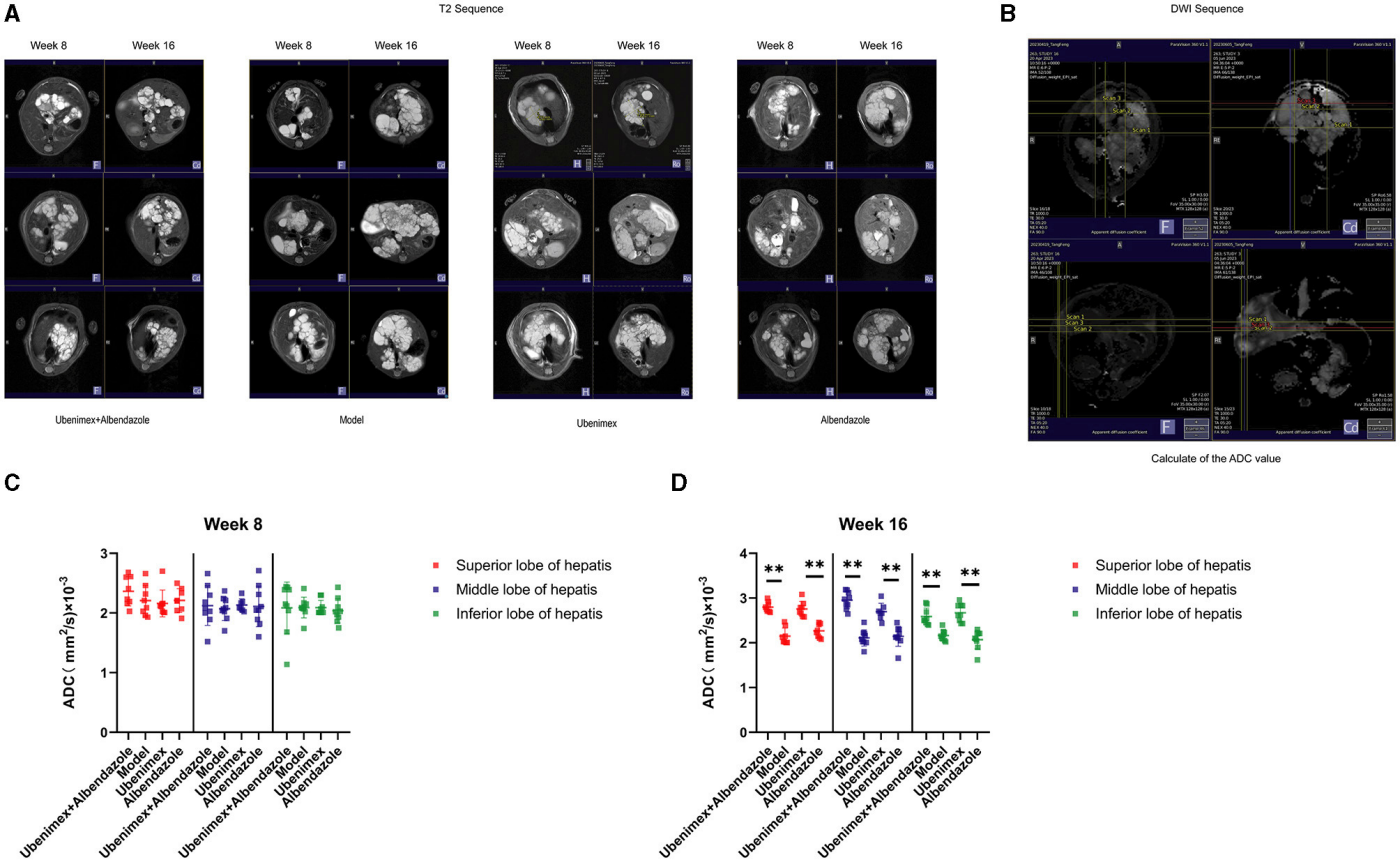
The MRI result in each group. **(A)** T2 scanning at week 8 and week 16. **(B)** DWI scanning. **(C, D)** The junctional area were measured to calculate the ADC values, and choice the superior, middle and inferior lobes of the liver DWI result to calculated at week 8 and week 16. ^**^P < 0.05.

All mice underwent MRI (including T1, T2 and DWI, which could indicate the anatomical position, *E. multilocularis* lesions, and the invasion of cysts and metacestodes in the liver, respectively). The T2 sequence results (Figure 1A) showed that at week 8, cysts were distributed throughout the liver in all mice with almost no difference in size and location. At week 16, the cysts grew rapidly and almost occupied the whole liver in the model group. In comparison, cyst growth was significantly inhibited in mice treated with both Ubenimex and Albendazole, but there was no obvious change in cyst size in mice treated with only Ubenimex or Albendazole. A dark dividing line was observed surrounding liver tissues in the combination group and the Ubenimex group, indicating that infiltration of cysts was inhibited.

The DWI sequence was obtained and the ADC value was calculated from the superior, middle and inferior lobes of the liver (Figure 1B). The results indicated that at week 8 (Figure 1C), there was no significant difference in the ADC values among the four groups (*P* > 0.05); while at week 16 (Figure 1D), the ADC values of the combination group and Ubenimex group were significantly higher than that of the model group and Albendazole group (*P* < 0.05), but there was no significant difference between the model group and the Albendazole group (*P* > 0.05).

**Figure 2:**
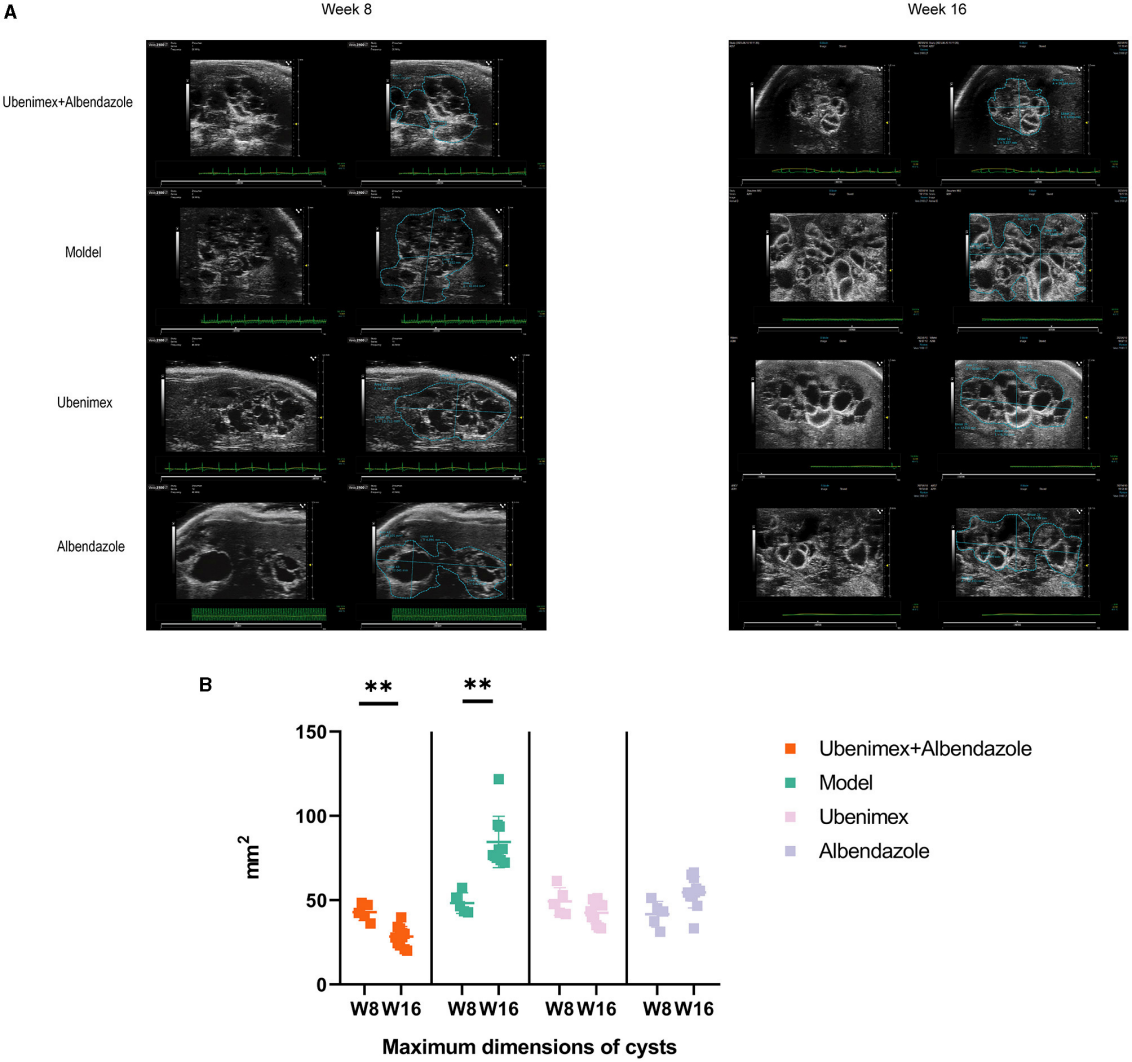
Ultrasonography. **(A)** Cysts in different groups and time points, at week 8 and week 16 to examine. **(B)** The maximum dimensions of cysts. ^**^P < 0.05.

The ultrasonic results showed that (Figure 2A) at week 8, many cysts of different sizes were closely packed in the liver and some cysts invaded into liver tissues in all mice; while at week 16, the cyst sizes were significantly increased in the model group (*P* < 0.05); significantly decreased in the combination group (P < 0.05), but remained almost unchanged in the Ubenimex group and Albendazole group (*P* > 0.05) (Figure 2B).

### Morphological and pathological results

**Figure 3:**
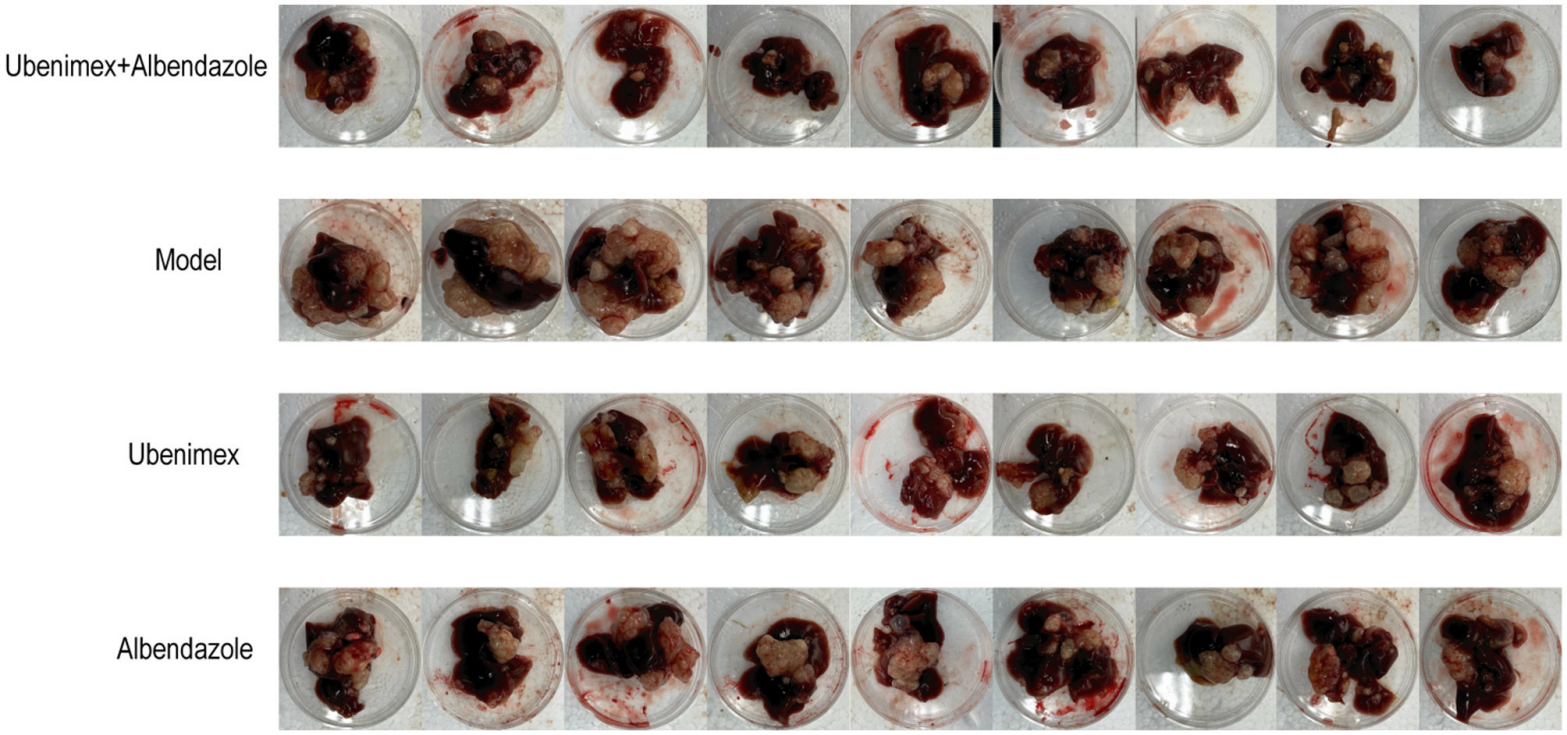
The morphological result: cysts of *metacestodes* in the liver at week 17.

All mice were sacrificed at week 17 and the cysts in the liver and peritoneum were excised and photographed. A large number of cysts of different sizes were observed in the model group and they were closely connected to liver tissues with no apparent demarcation, indicating an infiltrative growth pattern (Figure 3). However, the numbers and sizes of cysts were decreased in most mice treated with Ubenimex and Albendazole. The cysts were not tightly connected to liver tissues and some cysts could be easily peeled off, indicating that the combined treatment of Ubenimex and Albendazole inhibited the invasion of cysts. The same phenomenon was also observed in the Ubenimex group, but the sizes and numbers of cysts were not obviously changed. However, mice in the Albendazole group showed no obvious changes in the size, number and infiltration of cysts. Thus, the combined treatment of Ubenimex and Albendazole led to a substantial decrease in the size, number and infiltration of cysts in the liver.

**Figure 4:**
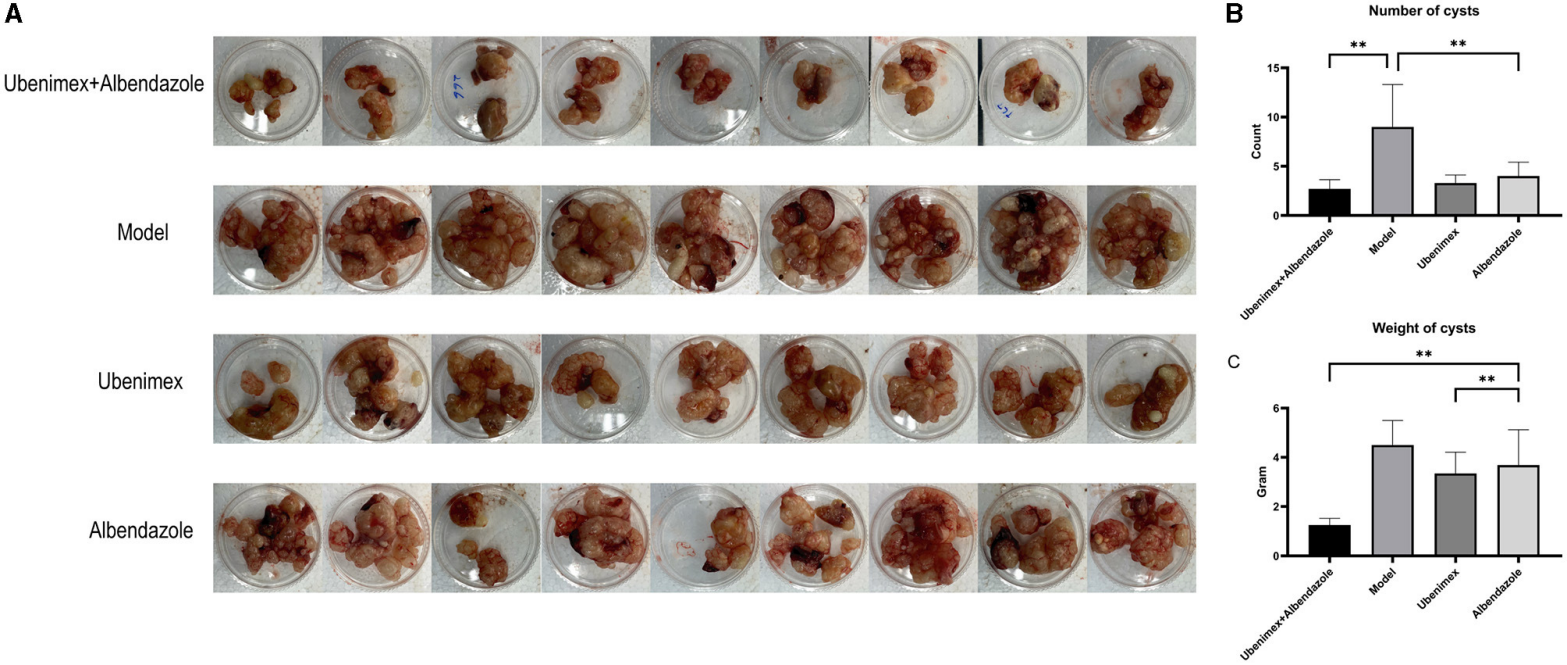
The cysts in abdomen. **(A)** The size and number of cysts at week 17. **(B)** The number of cysts in different groups. **(C)** The weight of cysts in different groups (**P < 0.05 v.s. Model group).

There was a significant difference in the number and weight of cysts collected from abdomen and liver of mice treated with different drugs (Figure 4A). Compared to the model group, the number (Figure 4B) of cysts was significantly decreased in the combination group, Albendazole group and Ubenimex group (*P* < 0.05); the weight (Figure 4C) was mostly significantly decreased in the combination group (*P* < 0.05); However, there was a significant difference in the weight of cysts between Albendazole group and Ubenimex group (*P* < 0.05). These results suggest that the combination of Ubenimex and Albendazole may be a viable treatment option for the infection of *E. multilocularis protoscoleces*.

**Figure 5:**
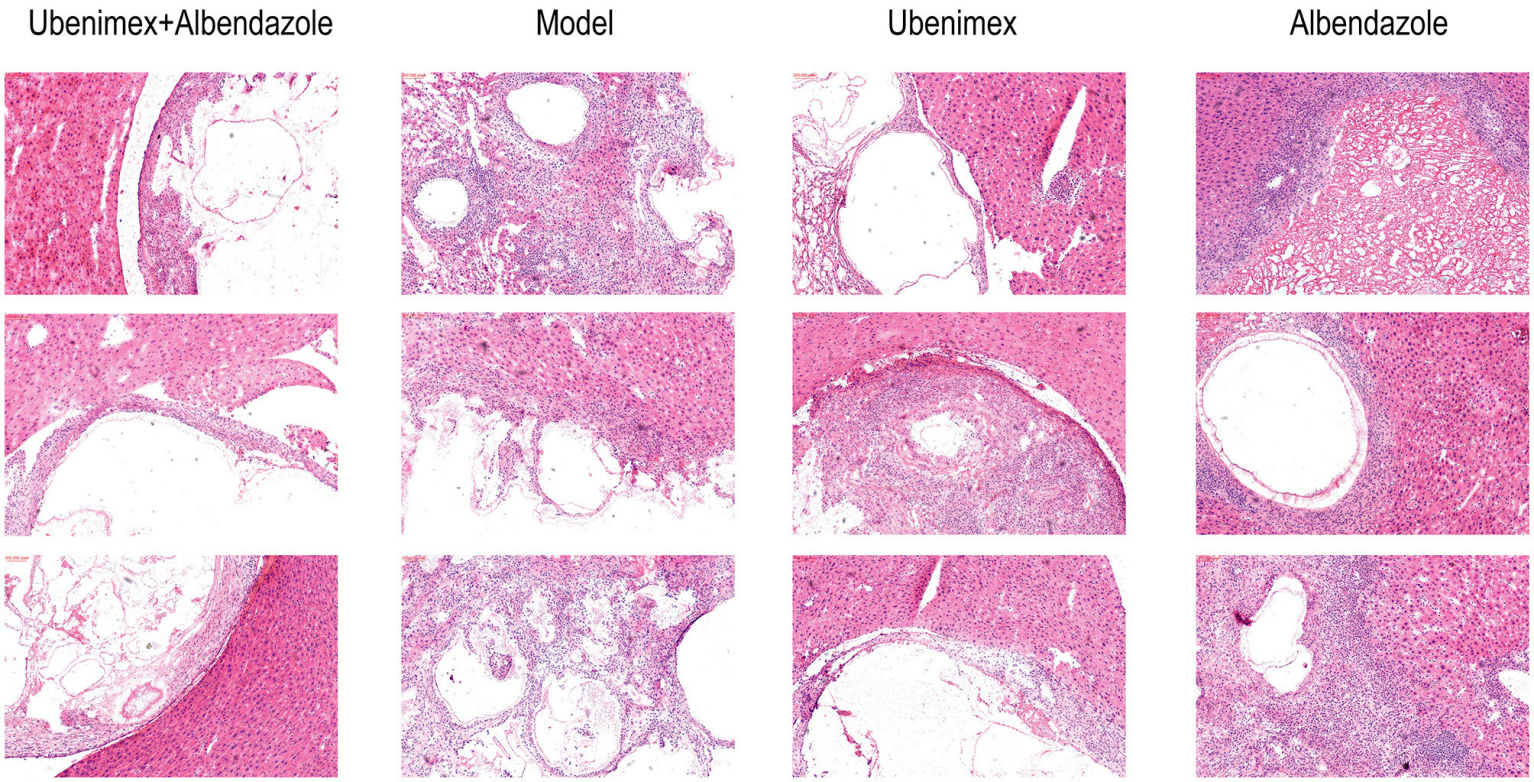
The Pathological result: histological sections after hematoxylin–eosin staining (100 × ).

The HE staining results revealed that there was a clear boundary between cysts and liver tissues in mice treated with Ubenimex and Albendazole, which indicated a non-infiltrative growth pattern. The germinal layer of the cysts was swollen and the number of metacestodes was reduced. Broken cysts were infiltrated by host inflammatory cells such as lymphocytes, neutrophils, and eosinophils, leading to collapse of the laminate and amorphous necrosis of cysts. However, mice in the Ubenimex group showed less host inflammatory cells in the cysts, less amorphous necrosis, and less swelling of the germinal layer. In the model group, a large number of cysts were observed in the liver with apparent vascular invasion and no clear boundary with surrounding liver tissues, indicating severe infiltrative growth. Clear laminated and germinal layers were observed; the cell density of the germinal layer was high and the number of metacestodes was increased. In the Albendazole group, the cysts also showed an infiltrative growth pattern, but the germinal layer in the cysts was mildly swollen and metacestodes disappeared. Proliferation of fibrous tissue was seen in some cysts as a red hyaline deformation, and there were a large number of host inflammatory cells surrounding the cysts (Figure 5).

**Figure 6:**
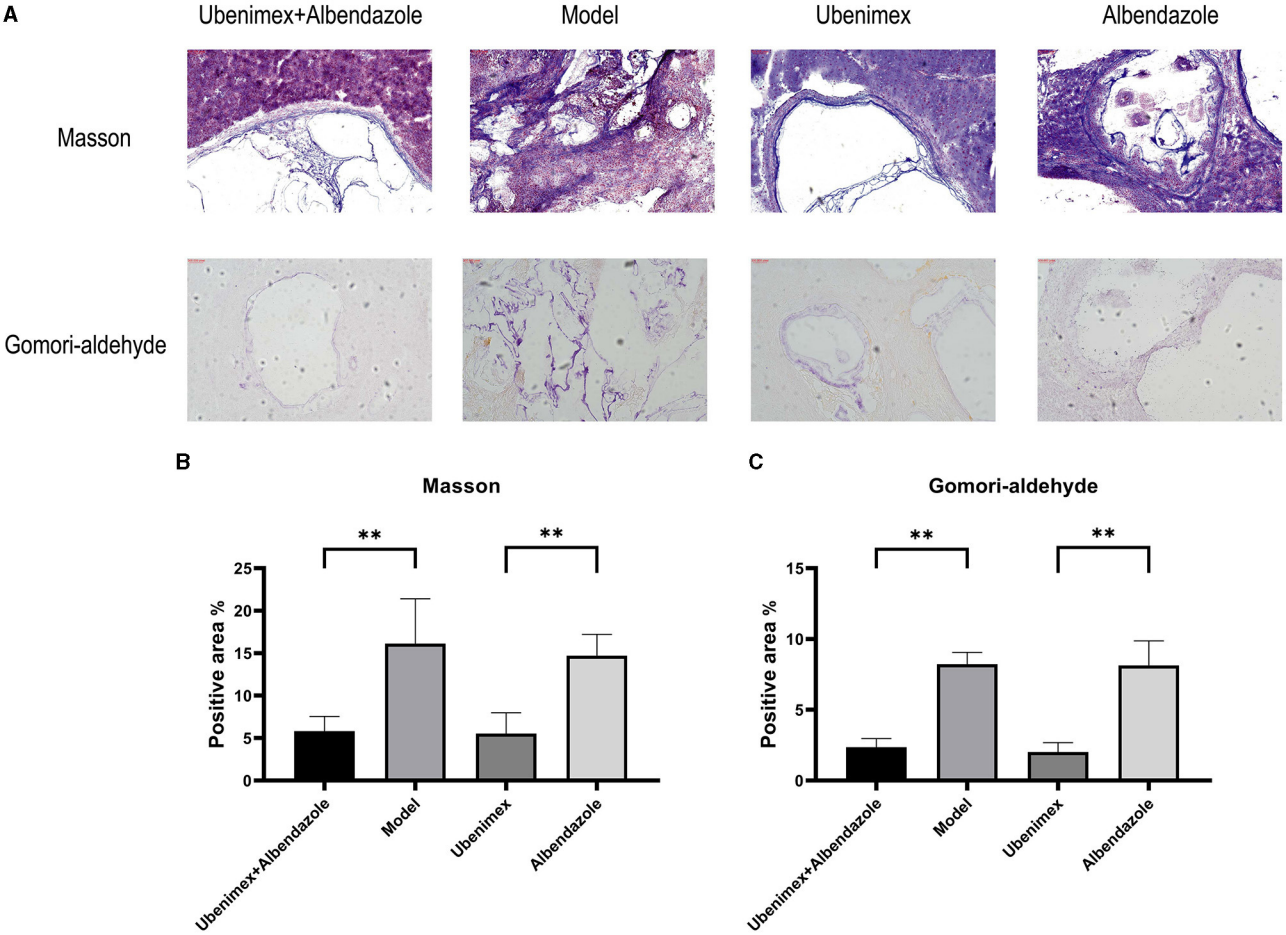
Histological sections. **(A)** Representative Masson- and Gomori-aldehyde-fuchsin-stained sections (100 × ); **(B, C)** Positive staining area (%) is presented in each image. The results are obtained from several images of 3 mice for each staining (**P < 0.05).

The Masson and Gomori-aldehyde-fuchsin staining results revealed that in the combination group and Ubenimex group, liver fibrosis and cyst invasion were significantly inhibited and the levels of collagenous and elastic fibers which were stained blue were decreased in the liver but increased in the cysts; while in the model group and Albendazole group, high levels of collagenous and elastic fibers were found in the liver and the cysts showed an infiltrative growth pattern (Figure 6A).

The positive levels of Masson–stained and Gomori-aldehyde-fuchsin–stained cysts were detected by ImageJ. The results (Figure 6B) showed that the levels of Masson-positive cysts of the combination group and Ubenimex group were significantly reduced compared to the model group and Albendazole group (*P* < 0.05), but there was no significant difference between model and Albendazole group or between combination and Ubenimex group. The same result was also observed for Gomori-Aldehyde-Fuchsin–positive cysts (Figure 6C).

### Liver functions

In this study, the levels of ALT, AST, TBIL, DBIL, ALB, ALP, and γ-GT in the serum were detected to determine liver damages of mice in each group. The results (Table 2) indicated that compared to the model group, the levels of ALT, AST, TBIL, DBIL, ALP, and γ-GT were significantly decreased in the combination group, Ubenimex group and Albendazole group. Compare to the blank group (healthy mice without any infection and treatment), the levels of AST, DBIL and ALB were significantly increased in the combination group; and the levels of ALT, AST, DBIL, ALP, and γ-GT were significantly increased in the Ubenimex group and Albendazole group.

**Table 2:** The test of liver function index.

	**Ubenimex + Albendazole**	**Model**	**Ubenimex**	**Albendazole**	**Control group**
ALT (U/L)	28.81 ± 3.98^*^	42.25 ± 2.40^#^	29.68 ± 5.69^*#^	32.20 ± 6.48^*#^	23.1 ± 2.04^*^
AST (U/L)	106.98 ± 4.19^*#^	133.30 ± 8.5^#^	103.93 ± 9.94^*#^	100.09 ± 9.56^*#^	83.09 ± 7.2^*^
TBIL (umol/L)	11.1 ± 2.36^*^	15.2 ± 2.9	10.59 ± 2.39^*^	10.89 ± 2.11^*^	8.68 ± 1.86^*^
DBIL (umol/L)	5.6 ± 0.78^*#^	8.47 ± 0.91^#^	6.71 ± 0.76^*#^	7.07 ± 0.68^*#^	4.35 ± 0.45^*^
ALB (g/L)	29.44 ± 2.73^#^	28.8 ± 2.02^#^	29.19 ± 1.7^#^	31.18 ± 1.07^*^	32.81 ± 6.96
ALP (U/L)	39.54 ± 6.96^*^	73.26 ± 8.34^#^	46.93 ± 7.93^*#^	50.9 ± 5.96^*#^	36.77 ± 1.07^*^
γ-GT (U/L)	0.92 ± 0.17^*#^	1.35 ± 0.21^#^	0.83 ± 0.32^*#^	0.9 ± 0.07^*#^	0.33 ± 0.07^*^

^*^Compare with Model group, P < 0.05.

^#^Compare with Control group, P < 0.05.

## Discussion

AE is a refractory parasitic disease and also a neglected parasitic zoonosis, as the parasite has high adaptability and immune escape capability (37) that make it difficult to clean, and it can infiltrate into other tissues and organizations in the host like a malignant tumor. In clinic, the single-drug therapy is of limited benefit and surgery is the only way to remove the parasites, but recurrence and metastasis are still common (38, 39). Liver transplantation is the only therapeutic strategy in end-stage patients (40). Albendazole is the only drug for the treatment of AE, but its effect is unsatisfactory. Therefore, there is a need for new therapeutic approaches to eliminate hydatid lesions and inhibit infiltration and fibrosis in the liver.

At present in clinical practice, only Albendazole and surgical operation be applied to treat AE, but has less curative effect, more than 20% of cases fail therapy (41), and beyond that, high dose and long-term medication with Albendazole may increase the toxic and side effect in host. So, many studies have focus on find a new therapy method, which can eliminate the hydatid lesions efficiency and less side effect in host. Liu et al. (31) found that, Thiacloprid, as one of insecticide which has favorable efficacy to treat AE *in vitro* and *in vivo*, and low mammalian toxicity to hosts, Thiacloprid can inhibited the acetylcholinesterase activity in hydatid *protoscoleces* and *metacestodes* that can damaged the germinal layer, but it still has some latent unknown side effect such as hypersensitivity need further research. Fabbri et al. (42) reported that the dichlorophen (DCP) and silica nanoparticles modified with DCP (NP-DCP) both demonstrated a time and dose-dependent *in vitro* effect against *protoscoleces*. Some new horizons of herbal drugs to cure CE are opening with every passing day (43), AE was no exception, some research reported that the Chinese traditional medicine-Crocin exert parasiticidal activity against *E. multilocularis in vitro* and *in vivo* (44); Jiang (45) indicated that a novel compound extracted from traditional Chinese medicine have efficacy of AE. Even though there's numerous research on innovate treatment AE, but still have insufficient, safety and efficient treatment method was essential, need long-term refinement and validation then can translate into clinical application.

In recent years more and more researches are also explore the conventional antiparasitic drugs' therapeutic potential, designed to increase efficacy and reduce toxic side effects, for instance Albendazole combination other drugs which was an effective way to treat AE (11–13). Therefore, in this study, a potential therapeutic strategy is to combine Ubenimex with Albendazole to reduce or inhibit the infiltrative and invasive growth of *E. multilocularis*.

We have previously found that vaccination with EM-LAP induced specific immune responses and inhibited the growth of *E. multilocularis*, and it is a potential therapeutic target for the treatment for AE (7, 8). LAP is an aminopeptidase from the M17 family, which is widely expressed in parasites and is an important metabolic enzyme that can digestive host tissues and then cause serious diseases (46). In our previous studies, we found that EM-LAP also allowed infiltrative growth of hydatid lesions in host liver. As a broad-spectrum inhibitor of EM-LAP, Ubenimex can reduce EM-LAP and inhibit the infiltrative growth of *E. multilocularis* (8), and thus it can act as a candidate drug for the treatment of AE. However, Ubenimex alone has little effect on the ontogeny and development of *E. multilocularis* but a better effect in inhibiting the invasion and infiltrative growth of hydatid lesions in liver. Thus, Ubenimex can be used in combination with other anthelmintic drugs to eliminate *E multilocularis* and hydatid lesions.

Ubenimex is one of LAP inhibitors that can reduce the activity of LAP, and therefore it can reduce the digestion of liver tissues, fibrogenesis and infiltrative growth (8, 47, 48). It has also reported that Ubenimex can reduce the invasiveness and damage of other parasites like *Leishmania*, Human liver *fluke, plasmodium* by inhibiting the LAP proteins levels (27, 49, 50). In some malignant tumors, Ubenimex can also inhibit the migration and invasion by alleviating the activity of the LAP (CD13)/NAB1/MAPK pathway (51–54).

Albendazole is a broad-spectrum anthelmintic which can inhibit multiple parasitic infections (55), but it is less effective for the infection of *E. multilocularis*. Many other studies have also explored how to enhance the efficacy of Albendazole in deworming (56, 57). Albendazole sulphoxide binds to parasite ß-microtubules, preventing them from aggregating into microtubules to disrupt the cascade of mid-stage cell division and ultimately leading to the death of individual cells and then the death of the parasite (58), Albendazole sulphoxide can also inhibit the glucose absorption of the parasite, leading to glycogen depletion, or inhibit the fumarate reductase system, blocking the production of ATP and therefore making it impossible for the parasite to survive and reproduce (59). However, Albendazole has low solubility in water and drug concentration in hydatid lesions (60), and different from Ubenimex, it has little inhibitory effect on hydatid infiltrative growth and invasion.

Single Ubenimex and Albendazole treatment has a limited therapeutic effect and could not inhibit the growth or invasion of cysts. In this study, the combination of Ubenimex and Albendazole can be an effective therapeutic approach for the treatment of AE in mice infected with *E. multilocularis*, which could reduce the growth, infiltration, invasion and fibrosis of hydatid cysts.

In this study, the combined use of two drugs demonstrated superior therapeutic effects compared to single drug administration, The potential reasons for this could be that, on one hand, both drugs participate in the parasite metabolism, but intervening from two different pathways, which can block the uptake of protein amino acids and glucose, making it difficult for the parasite to obtain nutrients from the host. On the other hand, the metabolic pathways blocked by the two drugs happen to be the pathways for the invasive growth of the parasite, significantly reducing the parasite's harm to the host. Therefore, the combined use of the drugs can exert a better synergistic effect. In addition, due to the different administration methods of the two drugs, the occurrence of unpredictable direct interactions between the two drugs is minimized, allowing each to achieve the desired effect to the greatest extent.

The two drugs act synergistically to provide an effective strategy for *E. multilocularis* infection and growth. However, neither single drug nor combined treatment led to significant changes in the liver function, indicating that these two drugs are relatively safe.

Nowadays, there have been numerous studies on the combined use of drugs to treat AE. However, these studies have primarily focused on enhancing the efficacy of Albendazole or increasing the blood concentration of Albendazole (61–63), without intervening in the infiltrative, fibrotic, and metastatic aspects of *E. multilocularis* infection. In conclusion, our study provides a new effective combined strategy for the treatment AE, which can not only reduce the growth of *E. multilocularis metacestodes* but can also inhibit the infiltration, invasion and fibrosis in host liver.

AE as one of regional public health issues that serious threat to the health of the ethnic minority areas population in China, the custom and living habits of ethnic minorities such as using no soap for cleaning dirty hands and eating raw food, are the main causes for the difficult to prophylaxis (64). For this reason, health education and propagating drug knowledge is an effective means of prevent the AE. Therefore, in this study we aim to come up with more effective drug combinations to treatment AE, not only reduce the growth of *E. multilocularis metacestodes*, but also inhibition of infiltration, invasion and fibrosis in host liver. On this account present the effective prevention and treatment measures of AE.

In this experiment, we focused on the therapeutic effect of drug combination. However, there still has some limitations. We did not investigate the mechanism of the combined drug therapy. In future studies, we will continue to explore the mechanism and pharmacological principles of combined drug therapy to optimize treatment strategies and further enhance therapeutic effects.

For the last few years, the parasitic disease was more and more paid attention, not only in *E. multilocularis* infection, but also other epidemic parasites. Meanwhile the conventional antiparasitic drugs occurrence anthelmintic resistance (65), such as Albendazole, due to the anthelmintic resistance, low solubility in water and less drug concentration in lesions, it was not exerted completely eliminate effect in some parasites infection. So, numerous researches were searching for a new therapeutic method, Velázquez-Antunez et al. (66) found that the secondary compounds of *Guazuma ulmifolia* leaves can inhibit the hatching of eggs of *Haemonchus contortus*; Al-Saeed et al. (67) reported that *Haloxylon salicornicum* leaves extract can exhibited a strong anthelmintic potential against haemonchosis *in vitro*. Thus, those methods can be developed as a novel drug for the treatment of some parasites. But antiparasitic drug development was a long process, need long-term refinement and validation then can translate into clinical application, so in future research, we will explore new Treatment methods and new drugs, so that increase therapeutic effect and decrease toxic-side effect and anthelmintic resistance.

## Conclusions

The combined treatment of Ubenimex and Albendazole can well inhibit the growth, development, invasion and fibrosis of cysts and protect the liver from damage by *E. multilocularis*. Thus, it is a potential candidate for the treatment of AE.

## Data availability statement

The original contributions presented in the study are included in the article/supplementary material, further inquiries can be directed to the corresponding authors.

## Ethics statement

All animal experiments were performed in compliance with the regulations of the Ministry of Science and Technology of China and approved by the Experimental Committee of Qinghai University (QHDX-2019-09). The study was conducted in accordance with the local legislation and institutional requirements.

## Author contributions

ZZ: Conceptualization, Data curation, Formal analysis, Methodology, Writing – original draft, Writing – review & editing. MH: Data curation, Formal analysis, Methodology, Writing – original draft. YM: Data curation, Writing – original draft. FT: Conceptualization, Writing – review & editing. R-LG: Conceptualization, Writing – original draft, Writing – review & editing.

## References

[1] Yang S Wu J Ding C Cui Y Zhou Y Li Y. Epidemiological features of and changes in incidence of infectious diseases in China in the first decade after the SARS outbreak: an observational trend study. Lancet Infect Dis. (2017) 17:716–25. 10.1016/S1473-3099(17)30227-X 28412150 PMC7164789

[2] Aydinli B Ozturk G Arslan S Kantarci M Tan O Ahiskalioglu A. Liver transplantation for alveolar echinococcosis in an endemic region. Liver Transpl. (2015) 21:1096–102. 10.1002/lt.24195 26074280

[3] Xu X Qian X Gao C Pang Y Zhou H Zhu L. Advances in the pharmacological treatment of hepatic alveolar echinococcosis: from laboratory to clinic. Front Microbiol. (2022) 13:953846. 10.3389/fmicb.2022.953846 36003932 PMC9393627

[4] Wen H Vuitton L Tuxun T Li J Vuitton DA Zhang W. Echinococcosis: advances in the 21st century. Clin Microbiolo Rev. (2019) 32:e00075-18. 10.1128/CMR.00075-18 30760475 PMC6431127

[5] Horton J. Albendazole for the treatment of echinococcosis. Fundam Clin Pharmacol. (2003) 17:205–12. 10.1046/j.1472-8206.2003.00171.x 12667231

[6] Xu K Ahan A A. new dawn in the late stage of alveolar echinococcosis “parasite cancer”. Med Hypotheses. (2020) 142:109735. 10.1016/j.mehy.2020.109735 32344283

[7] Wang L Wei W Zhou P Liu H Yang B Feng L. Enzymatic characteristics and preventive effect of leucine aminopeptidase against *Echinococcus multilocularis*. Acta Trop. (2021) 222:106066. 10.1016/j.actatropica.2021.106066 34303691

[8] Zhou Z Zhou P Mu Y Wang L Cao Z Dong S. Therapeutic effect on Alveolar echinococcosis by targeting EM-leucine aminopeptidase. Front Immunol. (2022) 13:1027500. 10.3389/fimmu.2022.1027500 36311709 PMC9614657

[9] Pensel P Paredes A Albani CM Allemandi D Sanchez Bruni S Palma SD. Albendazole nanocrystals in experimental alveolar echinococcosis: enhanced chemoprophylactic and clinical efficacy in infected mice. Vet Parasitol. (2018) 251:78–84. 10.1016/j.vetpar.2017.12.022 29426481

[10] Chai JY Jung BK Hong SJ. Albendazole and Mebendazole as anti-parasitic and anti-cancer agents: an update. Korean J Parasitol. (2021) 59:189–225. 10.3347/kjp.2021.59.3.189 34218593 PMC8255490

[11] Lopez LM Pensel PE Fabbri J Albani CM Elissondo N Gambino G. The combination of carvacrol and albendazole enhanced the efficacy of monotherapy in experimental alveolar echinococcosis. Acta Trop. (2022) 225:106198. 10.1016/j.actatropica.2021.106198 34688631

[12] Loos JA Coccimiglio M Nicolao MC Rodrigues CR Cumino AC. Metformin improves the therapeutic efficacy of low-dose albendazole against experimental alveolar echinococcosis. Parasitology. (2022) 149:138–44. 10.1017/S0031182021001633 35184788 PMC11010535

[13] Hu C Zhang F Fan H. Improvement of the bioavailability and anti-hepatic alveolar echinococcosis effect of albendazole-isethionate/hypromellose acetate succinate (HPMC-AS) complex. Antimicrob Agents Chemother. (2021) 65:e0223320. 10.1128/AAC.02233-20 33875425 PMC8218690

[14] Wu M Yan M Xu J Liang Y Gu X Xie Y. Expression, tissue localization and serodiagnostic potential of *Echinococcus granulosus* leucine aminopeptidase. Int J Mol Sci. (2018) 19:1063. 10.3390/ijms19041063 29614002 PMC5979522

[15] Lee YR Na BK Moon EK Song SM Joo SY Kong HH. Essential role for an M17 leucine aminopeptidase in encystation of *Acanthamoeba castellanii*. PLoS ONE. (2015) 10:e0129884. 10.1371/journal.pone.0129884 26075721 PMC4468156

[16] Checa J Salazar C Goyeche A Rivera M Silveira F Maggioli G. A promising new target to control fasciolosis: *Fasciola hepatica* leucine aminopeptidase 2. Vet Parasitol. (2023) 320:109959. 10.1016/j.vetpar.2023.109959 37329826

[17] Edgar RCS Siddiqui G Hjerrild K Malcolm TR Vinh NB Webb CT. Genetic and chemical validation of *Plasmodium falciparum* aminopeptidase PfA-M17 as a drug target in the hemoglobin digestion pathway. eLife. (2022) 11:e80813. 10.7554/eLife.80813 36097817 PMC9470162

[18] Gardiner DL Trenholme KR Skinner-Adams TS Stack CM Dalton JP. Overexpression of leucyl aminopeptidase in *Plasmodium falciparum* parasites. Target for the antimalarial activity of bestatin. J Biol Chem. (2006) 281:1741–5. 10.1074/jbc.M508955200 16286469

[19] Skinner-Adams TS Peatey CL Anderson K Trenholme KR Krige D Brown CL. The aminopeptidase inhibitor CHR-2863 is an orally bioavailable inhibitor of murine malaria. Antimicrob Agents Chemother. (2012) 56:3244–9. 10.1128/AAC.06245-11 22450967 PMC3370795

[20] Cunningham E Drag M Kafarski P Bell A. Chemical target validation studies of aminopeptidase in malaria parasites using alpha-aminoalkylphosphonate and phosphonopeptide inhibitors. Antimicrob Agents Chemother. (2008) 52:3221–8. 10.1128/AAC.01327-07 18458130 PMC2533478

[21] Mathew R Wunderlich J Thivierge K Cwiklinski K Dumont C Tilley L. Biochemical and cellular characterisation of the *Plasmodium falciparum* M1 alanyl aminopeptidase (PfM1AAP) and M17 leucyl aminopeptidase (PfM17LAP). Sci Rep. (2021) 11:2854. 10.1038/s41598-021-82499-4 33536500 PMC7858622

[22] Yue K Hou X Jia G Zhang L Zhang J Tan L. Design, synthesis and biological evaluation of hybrid of ubenimex-fluorouracil for hepatocellular carcinoma therapy. Bioorg Chem. (2021) 116:105343. 10.1016/j.bioorg.2021.105343 34544027

[23] Yoneda J Saiki I Fujii H Abe F Kojima Y Azuma I. Inhibition of tumor invasion and extracellular matrix degradation by ubenimex (bestatin). Clin Exp Metastasis. (1992) 10:49–59. 10.1007/BF00163576 1733647

[24] Yamashita M Wada H Eguchi H Ogawa H Yamada D Noda T. A CD13 inhibitor, ubenimex, synergistically enhances the effects of anticancer drugs in hepatocellular carcinoma. Int J Oncol. (2016) 49:89–98. 10.3892/ijo.2016.3496 27121124 PMC4902077

[25] Guo Q Jing FJ Xu W Li X Li X Sun JL. Ubenimex induces autophagy inhibition and EMT suppression to overcome cisplatin resistance in GC cells by perturbing the CD13/EMP3/PI3K/AKT/NF-κB axis. Aging. (2019) 12:80–105. 10.18632/aging.102598 31895687 PMC6977684

[26] Dalal S Klemba M. Roles for two aminopeptidases in vacuolar hemoglobin catabolism in Plasmodium falciparum. J Biol Chem. (2007) 282:35978–87. 10.1074/jbc.M703643200 17895246

[27] Ariefta NR Pagmadulam B Hatano M Ikeda N Isshiki K Matoba K. Antiplasmodial activity evaluation of a bestatin-related aminopeptidase inhibitor, phebestin. Antimicrob Agents Chemother. (2023) 67:e0160622. 10.1128/aac.01606-22 37314349 PMC10353437

[28] Horton J. Albendazole: a broad spectrum anthelminthic for treatment of individuals and populations. Curr Opin Infect Dis. (2002) 15:599–608. 10.1097/00001432-200212000-00008 12821837

[29] Fabbri J Pensel PE Albani CM Lopez LM Simonazzi A Bermudez JM. Albendazole solid dispersions against alveolar echinococcosis: a pharmacotechnical strategy to improve the efficacy of the drug. Parasitology. (2020) 147:1026–31. 10.1017/S0031182020000670 32338226 PMC10317690

[30] Abulaihaiti M Wu XW Qiao L Lv HL Zhang HW Aduwayi N. Efficacy of albendazole-chitosan microsphere-based treatment for alveolar Echinococcosis in mice. PLoS Negl Trop Dis. (2015) 9:e0003950. 10.1371/journal.pntd.0003950 26352932 PMC4564103

[31] Liu C Fan H Ma J Ma L Ge RL. *In vitro* and *in vivo* efficacy of thiacloprid against *Echinococcus multilocularis*. Parasit Vectors. (2021) 14:450. 10.1186/s13071-021-04952-7 34488852 PMC8419995

[32] Li R Yang Q Guo L Feng L Wang W Liu K. Immunological features and efficacy of the recombinant subunit vaccine LTB-EMY162 against *Echinococcus multilocularis* metacestode. Appl Microbiol Biotechnol. (2018) 102:2143–54. 10.1007/s00253-018-8771-5 29354854

[33] Lis M Szczypka M Suszko A Obmińska-Mrukowicz B. The effects of bestatin on humoral response to sheep erythrocytes in non-treated and cyclophosphamide-immunocompromised mice. Immunopharmacol Immunotoxicol. (2013) 35:133–8. 10.3109/08923973.2012.719524 22957713

[34] Hossain A Heron D Davenport I Huckaba T Graves R Mandal T. Protective effects of bestatin in the retina of streptozotocin-induced diabetic mice. Exp Eye Res. (2016) 149:100–6. 10.1016/j.exer.2016.06.016 27344955 PMC5499666

[35] Zhu L Kuang X Zhang G Liang L Liu D Hu B. Albendazole induces immunotherapy response by facilitating ubiquitin-mediated PD-L1 degradation. J Immunother Cancer. (2022) 10:e003819. 10.1136/jitc-2021-003819 35577504 PMC9115032

[36] Jhan KY Cheng CJ Chou CJ Jung SM Lai GJ Chen KY. Improvements of cognitive functions in mice heavily infected by *Angiostrongylus cantonensis* after treatment with albendazole, dexamethasone, or co-therapy. J Microbiol Immunol Infect. (2022) 55:935–45. 10.1016/j.jmii.2022.04.004 35484079

[37] Zhang C Wang H Li J Hou X Li L Wang W. Involvement of TIGIT in natural killer cell exhaustion and immune escape in patients and mouse model with liver *Echinococcus multilocularis* Infection. Hepatology. (2021) 74:3376–93. 10.1002/hep.32035 34192365

[38] Atanasov G Benckert C Thelen A Tappe D Frosch M Teichmann D. Alveolar echinococcosis-spreading disease challenging clinicians: a case report and literature review. World J Gastroenterol. (2013) 19:4257–61. 10.3748/wjg.v19.i26.4257 23864792 PMC3710431

[39] Kaethner M Epping K Bernthaler P Rudolf K Thomann I Leitschuh N. Transforming growth factor-β signalling regulates protoscolex formation in the *Echinococcus multilocularis* metacestode. Front Cell Infect Microbiol. (2023) 13:1153117. 10.3389/fcimb.2023.1153117 37033489 PMC10073696

[40] Kamiyama T. Recent advances in surgical strategies for alveolar echinococcosis of the liver. Surg Today. (2020) 50:1360–7. 10.1007/s00595-019-01922-6 31768657

[41] Du C Liu Z Yang X Yan L Li B Wen T. Hepatectomy for patients with alveolar echinococcosis: long-term follow-up observations of 144 cases. Int J Surg. (2016) 35:147–52. 10.1016/j.ijsu.2016.09.094 27693514

[42] Fabbri J Pensel PE Albani CM Arce VB Mártire DO Elissondo MC. Drug repurposing for the treatment of alveolar echinococcosis: *in vitro* and *in vivo* effects of silica nanoparticles modified with dichlorophen. Parasitology. (2019) 146:1620–30. 10.1017/S0031182019001057 31397256

[43] Alvi MA Khan S Ali RMA Qamar W Saqib M Faridi NY. Herbal medicines against hydatid disease: a systematic review (2000-2021). Life. (2022) 12:676. 10.3390/life12050676 35629345 PMC9145516

[44] Liu C Fan H Guan L Ge RL Ma L. *In vivo* and *in vitro* efficacy of crocin against *Echinococcus multilocularis*. Parasit Vectors. (2021) 14:364. 10.1186/s13071-021-04866-4 34256821 PMC8278753

[45] Jiang C. Experimental study on a novel compound extracted from Traditional Chinese Medicine for treatment of alveolar echinococcosis. Chin Med J. (2002) 115:1576–8. 12490115

[46] McCarthy E Stack C Donnelly SM Doyle S Mann VH Brindley PJ. Leucine aminopeptidase of the human blood flukes, *Schistosoma mansoni* and *Schistosoma japonicum*. Int J Parasitol. (2004) 34:703–14. 10.1016/j.ijpara.2004.01.008 15111092

[47] Harbut MB Velmourougane G Dalal S Reiss G Whisstock JC Onder O. Bestatin-based chemical biology strategy reveals distinct roles for malaria M1- and M17-family aminopeptidases. Proc Natl Acad Sci U S A. (2011) 108:E526–34. 10.1073/pnas.1105601108 21844374 PMC3161592

[48] Singh AK Singh R Tomar D Pandya CD Singh R. The leucine aminopeptidase of *Staphylococcus aureus* is secreted and contributes to biofilm formation. Int J Infect Dis. (2012) 16:e375–81. 10.1016/j.ijid.2012.01.009 22410279

[49] Hassan AHE Mahmoud K Phan TN Shaldam MA Lee CH Kim YJ. Bestatin analogs-4-quinolinone hybrids as antileishmanial hits: design, repurposing rational, synthesis, *in vitro* and *in silico* studies. Eur J Med Chem. (2023) 250:115211. 10.1016/j.ejmech.2023.115211 36827952

[50] Khampoosa P Jones MK Lovas EM Piratae S Kulsuntiwong J Prasopdee S. Egg-hatching mechanism of human liver fluke, opisthorchis viverrini: a role for leucine aminopeptidases from the snail host, bithynia siamensis goniomphalos. J Parasitol. (2018) 104:388–97. 10.1645/16-125 29616885

[51] Liu X Guo Q Jing F Zhou C Xiu T Shi Y. Ubenimex suppresses the ability of migration and invasion in gastric cancer cells by alleviating the activity of the CD13/NAB1/MAPK pathway. Cancer Manag Res. (2021) 13:4483–95. 10.2147/CMAR.S300515 34113174 PMC8187004

[52] Wang X Niu Z Jia Y Cui M Han L Zhang Y. Ubenimex inhibits cell proliferation, migration and invasion by inhibiting the expression of APN and inducing autophagic cell death in prostate cancer cells. Oncol Rep. (2016) 35:2121–30. 10.3892/or.2016.4611 26846372

[53] Wan J Ling XA Wang J Ding GG Wang X. Inhibitory effect of Ubenimex combined with fluorouracil on multiple drug resistance and P-glycoprotein expression level in non-small lung cancer. J Cell Mol Med. (2020) 24:12840–7. 10.1111/jcmm.15875 32945069 PMC7687002

[54] Tsukamoto H Shibata K Kajiyama H Terauchi M Nawa A Kikkawa F. Aminopeptidase N (APN)/CD13 inhibitor, Ubenimex, enhances radiation sensitivity in human cervical cancer. BMC Cancer. (2008) 8:74. 10.1186/1471-2407-8-74 18366676 PMC2289833

[55] Babják M Königová A Burcáková L Komáromyová M Dolinská MU Várady M. Assessing the efficacy of Albendazole against *Fasciola hepatica* in naturally infected cattle by *in vivo* and *in vitro* methods. Vet Sci. (2021) 8:249. 10.3390/vetsci8110249 34822622 PMC8618507

[56] Movahedi F Li L Gu W Xu ZP. Nanoformulations of albendazole as effective anticancer and antiparasite agents. Nanomedicine. (2017) 12:2555–74. 10.2217/nnm-2017-0102 28954575

[57] da Silva Santana RC Prudente TP de Sousa Guerra CH de Lima NF de Souza Lino Junior R Vinaud MC. Albendazole - ivermectin combination decreases inflammation in experimental neurocysticercosis. Exp Parasitol. (2023) 251:108568. 10.1016/j.exppara.2023.108568 37327965

[58] Jacob J Siraj MA Steel A Tan G Jarvi S. Evaluation of the mechanism of action of albendazole on adult rat lungworm (*Angiostrongylus cantonensis*). Exp Parasitol. (2022) 242:108355. 10.1016/j.exppara.2022.108355 35988809

[59] Albanese G Venturi C. Albendazole: a new drug for human parasitoses. Dermatol Clin. (2003) 21:283–90. 10.1016/S0733-8635(02)00085-2 12757251

[60] Hu C Qin M Zhang F Gao R Gan X Du T. Improvement of antialveolar echinococcosis efficacy of novel albendazole-bile acids derivatives with enhanced oral bioavailability. PLoS Negl Trop Dis. (2023) 17:e0011031. 10.1371/journal.pntd.0011031 36595544 PMC9838834

[61] Stettler M Rossignol JF Fink R Walker M Gottstein B Merli M. Secondary and primary murine alveolar echinococcosis: combined albendazole/nitazoxanide chemotherapy exhibits profound anti-parasitic activity. Int J Parasitol. (2004) 34:615–24. 10.1016/j.ijpara.2004.01.006 15064126

[62] Enkai S Kouguchi H Inaoka DK Irie T Yagi K Kita K. *In vivo* efficacy of combination therapy with albendazole and atovaquone against primary hydatid cysts in mice. Eur J Clin Microbiol Infect Dis. (2021) 40:1815–20. 10.1007/s10096-021-04230-5 33770336 PMC8346398

[63] Xin Q Lv W Xu Y Luo Y Zhao C Wang B. 2-Deoxy-D-glucose and combined 2-Deoxy-D-glucose/albendazole exhibit therapeutic efficacy against *Echinococcus granulosus* protoscoleces and experimental alveolar echinococcosis. PLoS Negl Trop Dis. (2022) 16:e0010618. 10.1371/journal.pntd.0010618 35849619 PMC9333451

[64] McManus DP Zhang W Li J Bartley PB. Echinococcosis. Lancet. (2003) 362:1295–304. 10.1016/S0140-6736(03)14573-4 14575976

[65] Qamar W. and Alkheraije KA. Anthelmintic resistance in *Haemonchus contortus* of sheep and goats from Asia–a review of *in vitro* and *in vivo* studies. Pak Vet J. (2023) 43:376–87.

[66] Velázquez-Antunez JO-PJ Olmedo-Juárez A Rojas-Hernandez S Villa-Mancera A Romero-Rosales T Zamilpa A. Biological activity of the secondary compounds of *Guazuma ulmifolia* leaves to inhibit the hatching of eggs of *Haemonchus contortus*. Pak Vet J. (2023) 43:55–60.

[67] Al-Saeed FA Ismael Bamarni SS Iqbal KJ Rehman T Faruk AZ Mahmood S. *In vitro* anthelmintic efficacy of *Haloxylon salicornicum* leaves extract using adult *Heamonchus contortus* worms. Pak Vet J. (2023) 43:91–6. 10.29261/pakvetj/2022.091

